# Supplementary figures and images for: Filling gaps in PM2.5 time series: A broad evaluation from statistical to advanced neural network models
Source: PLoS One. 2025 Aug 14;20(8):e0330211. doi: 10.1371/journal.pone.0330211 (PMC12352854; doi:10.1371/journal.pone.0330211)

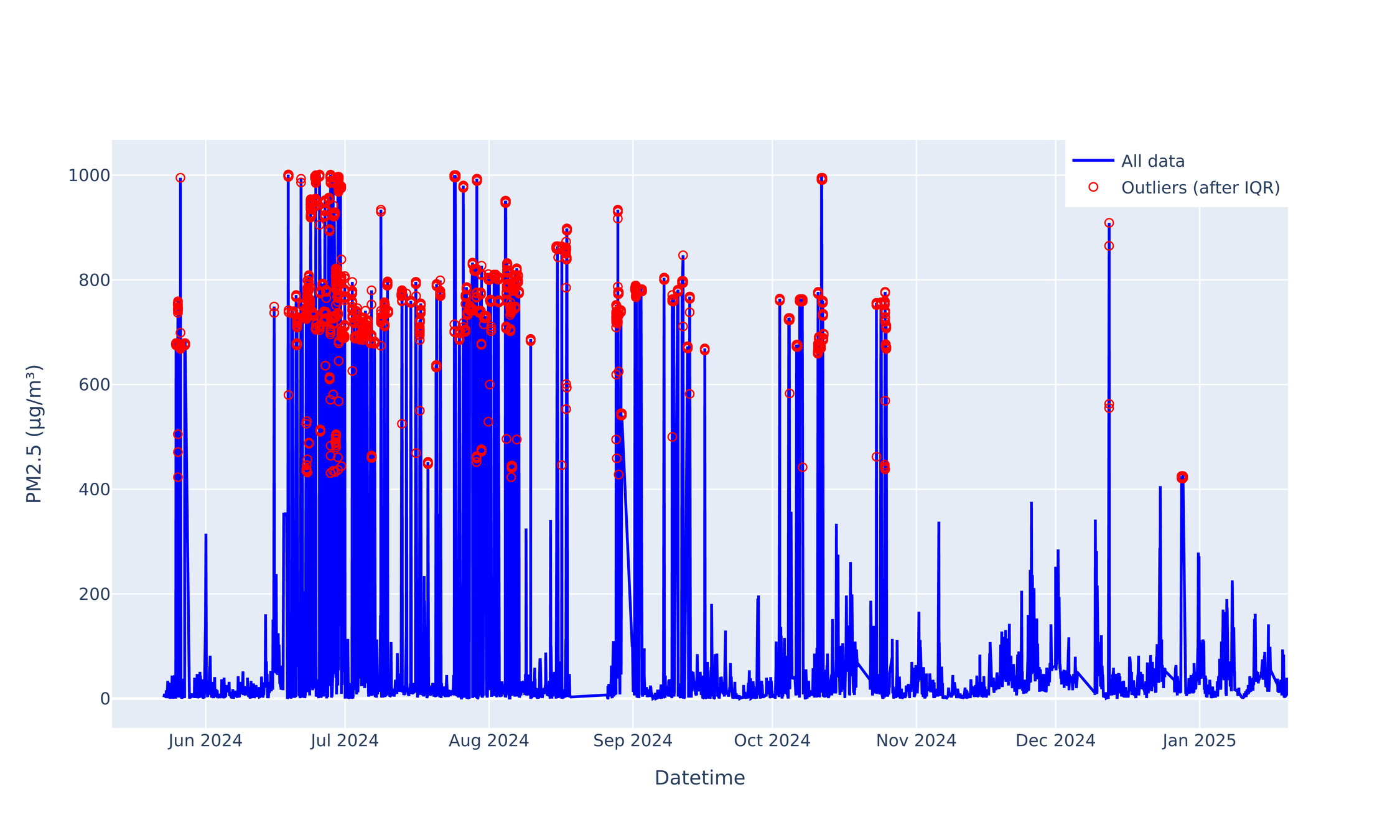

Supplement: S2 File — (ZIP) [file pone.0330211.s002.zip › Fig1_outliers.tif]

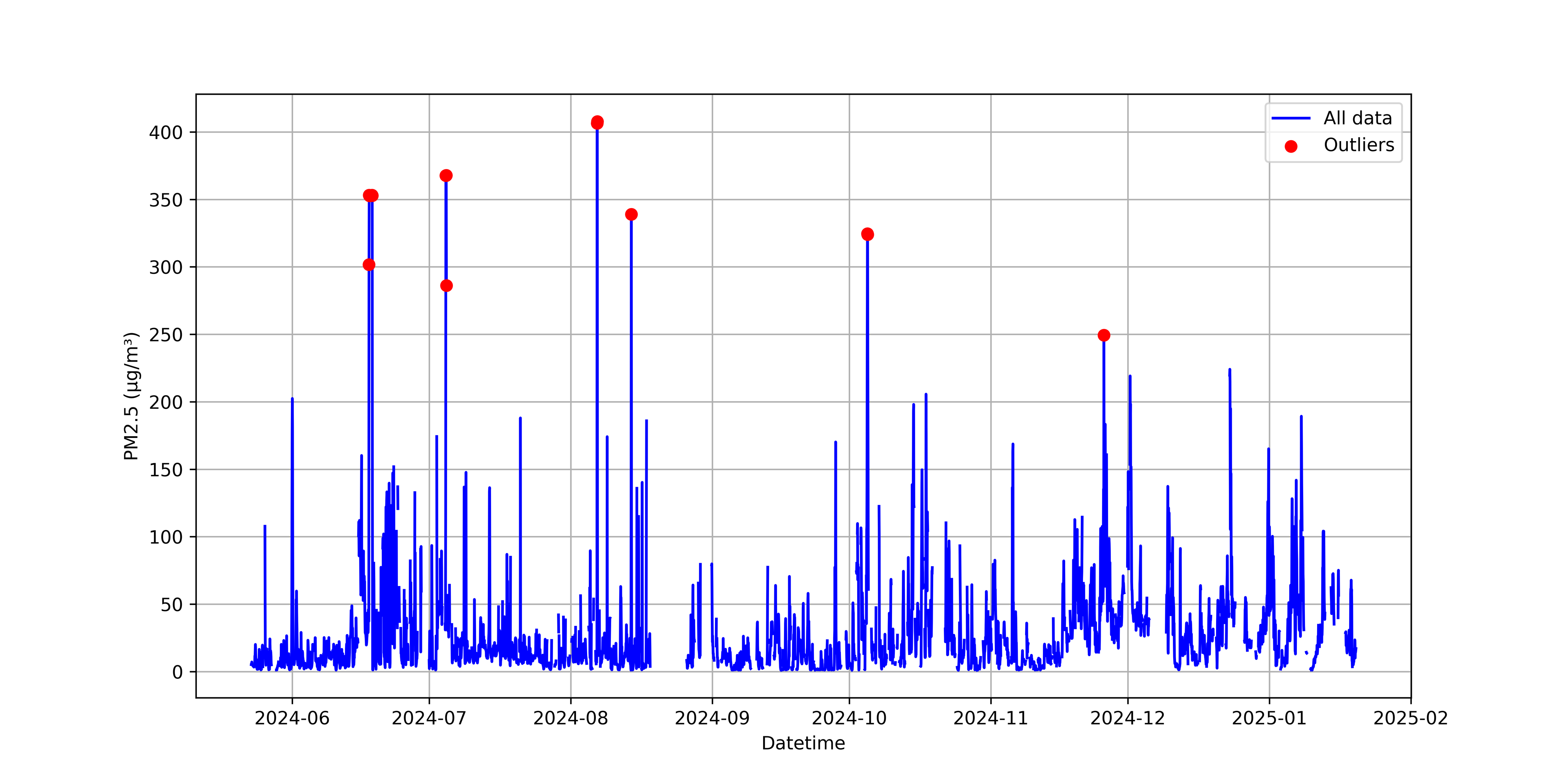

Supplement: S2 File — (ZIP) [file pone.0330211.s002.zip › Fig2_secondary_outliers_hourly.tif]

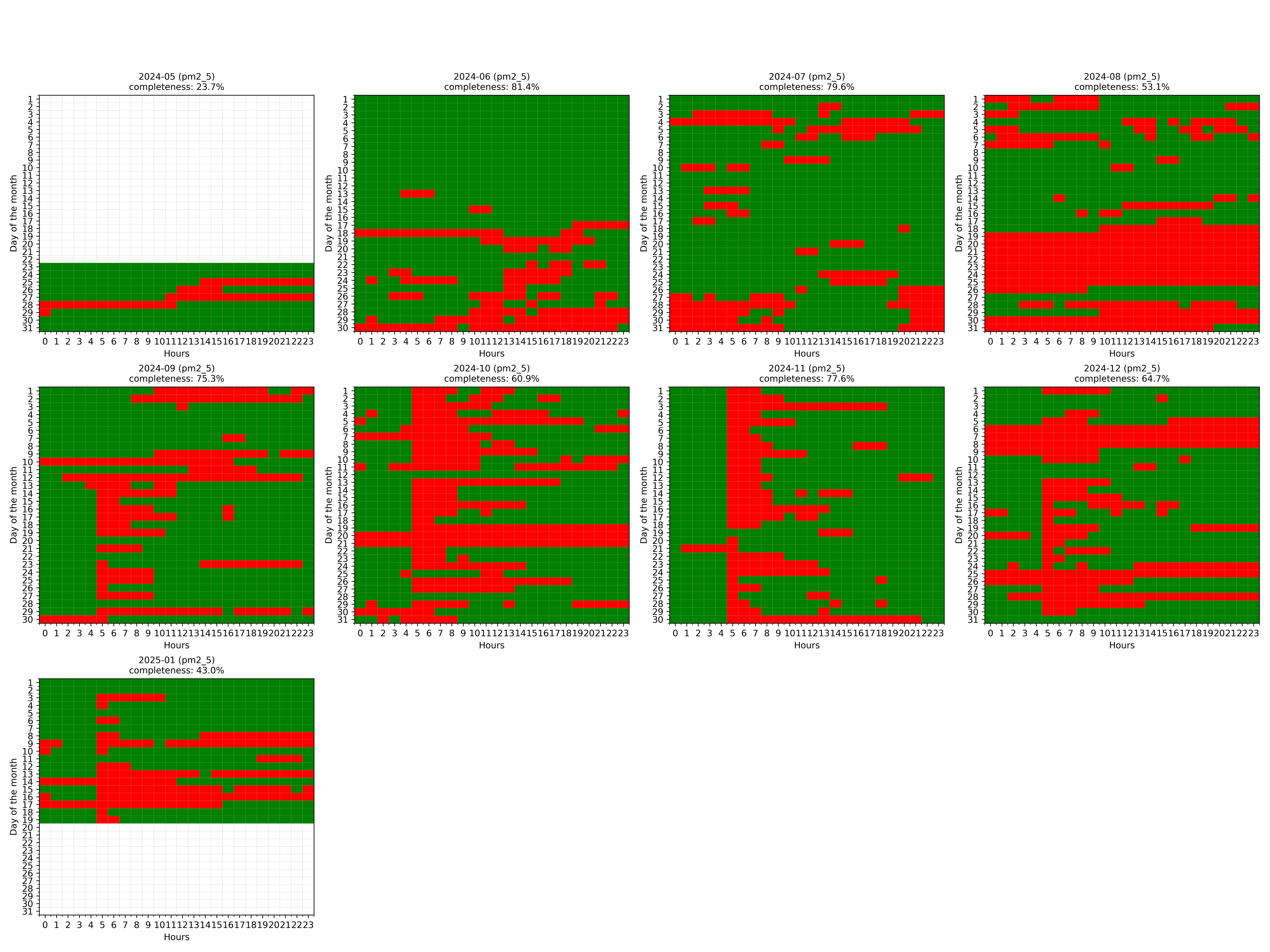

Supplement: S2 File — (ZIP) [file pone.0330211.s002.zip › Fig3_hourly_data_completeness_before.tif]

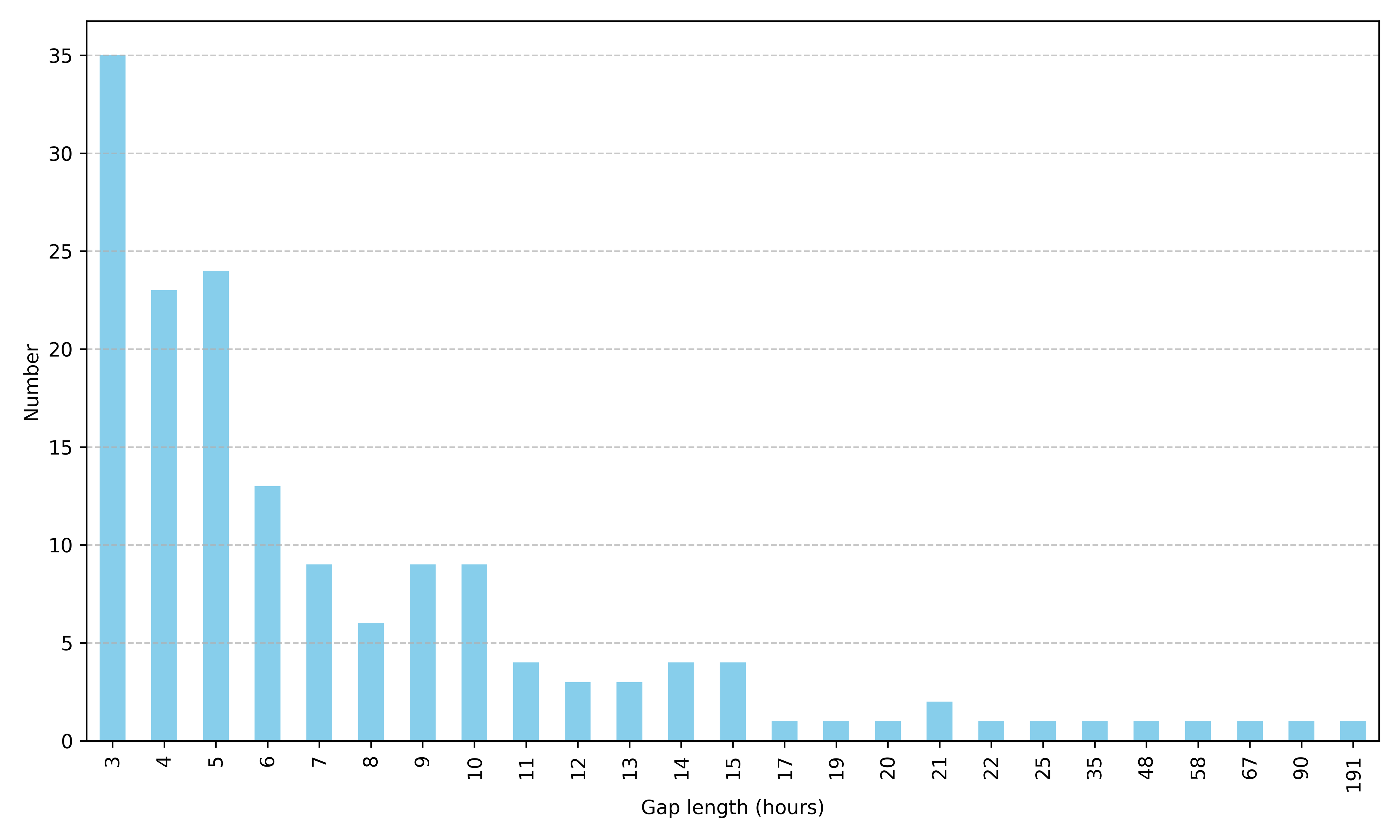

Supplement: S2 File — (ZIP) [file pone.0330211.s002.zip › Fig4_gap_distribution.tif]

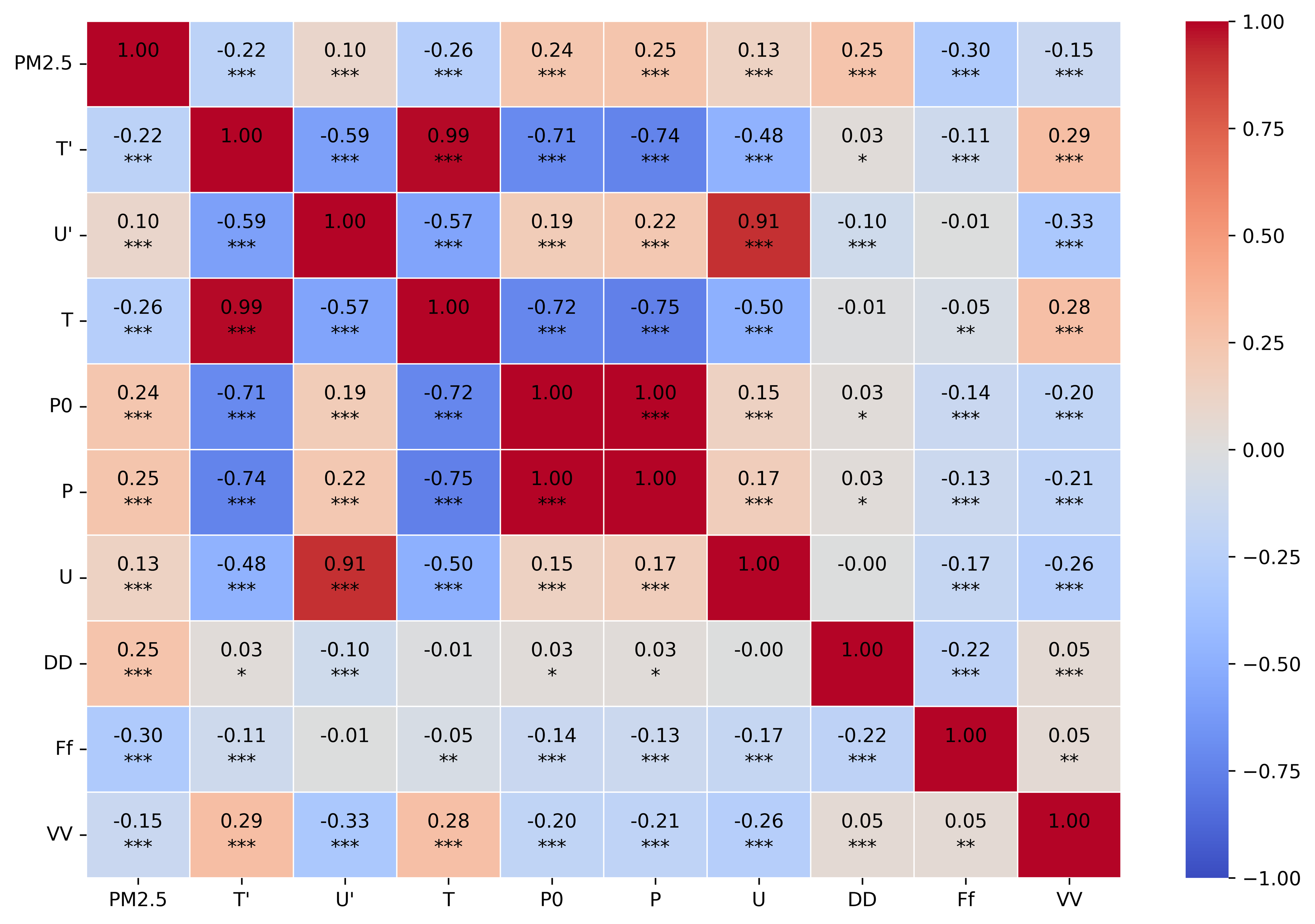

Supplement: S2 File — (ZIP) [file pone.0330211.s002.zip › Fig5_correlation_matrix_params.tif]

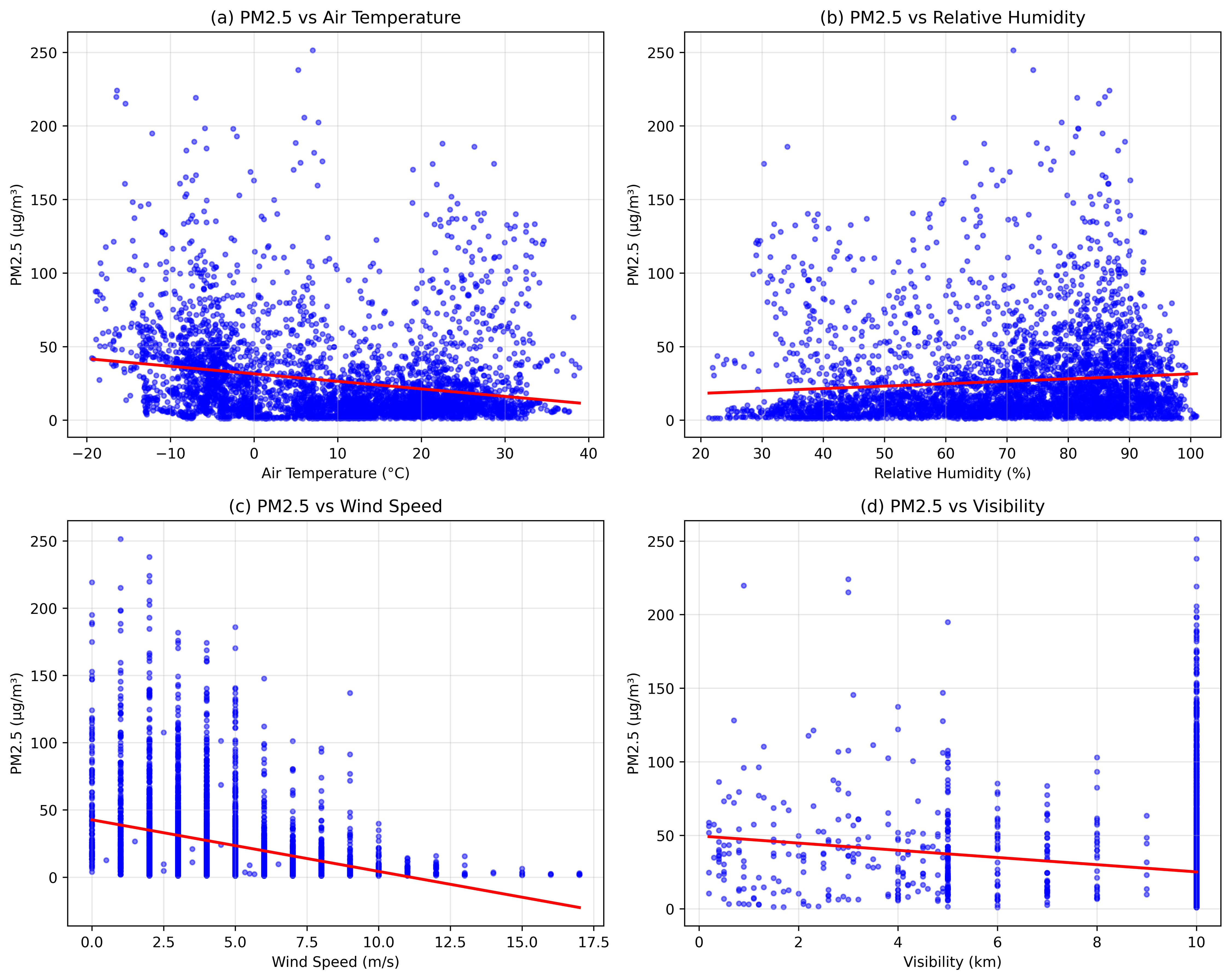

Supplement: S2 File — (ZIP) [file pone.0330211.s002.zip › Fig6_pm25_meteo_relationships.tif]

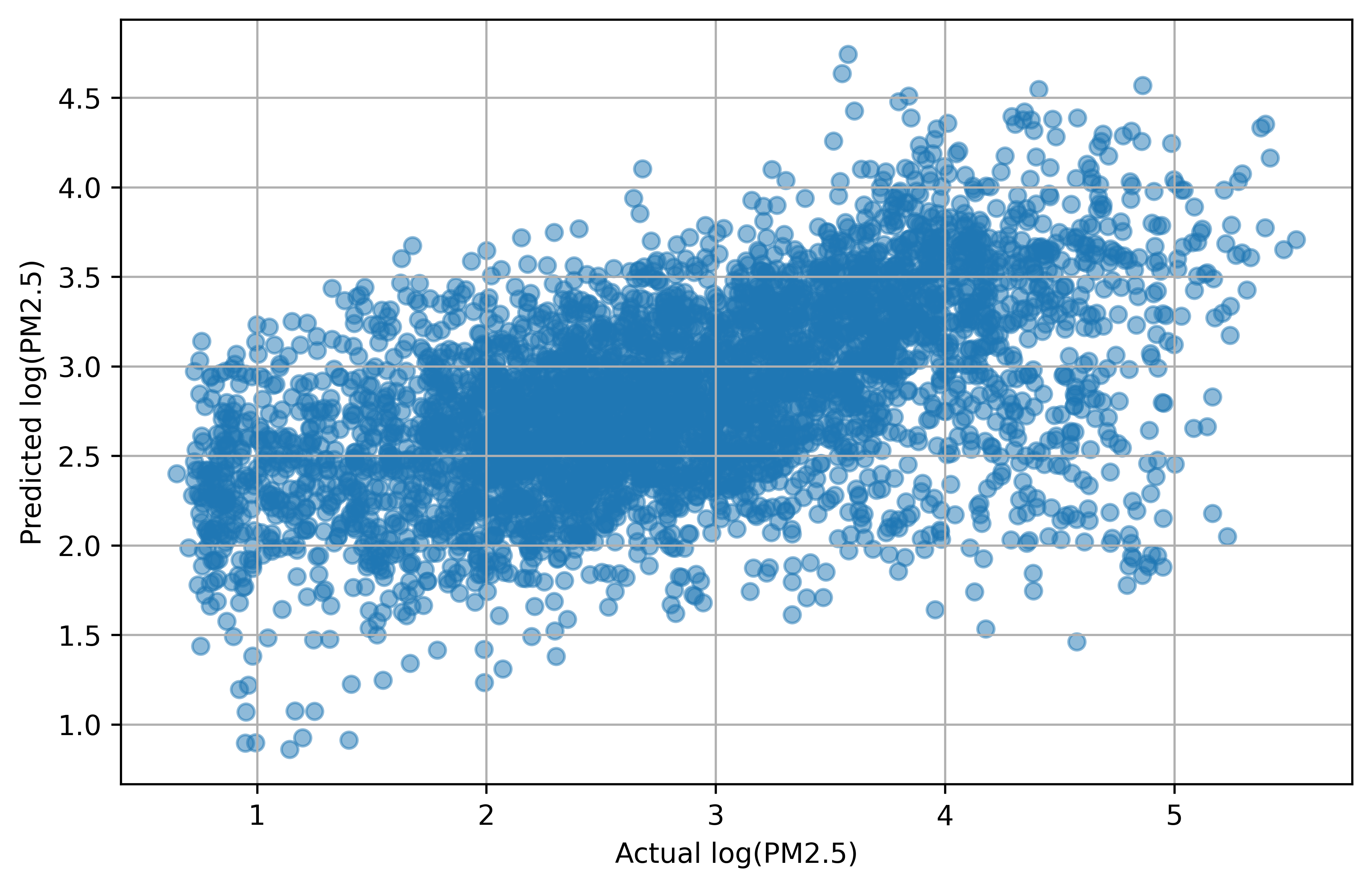

Supplement: S2 File — (ZIP) [file pone.0330211.s002.zip › Fig7_Log_reg_predictions.tif]

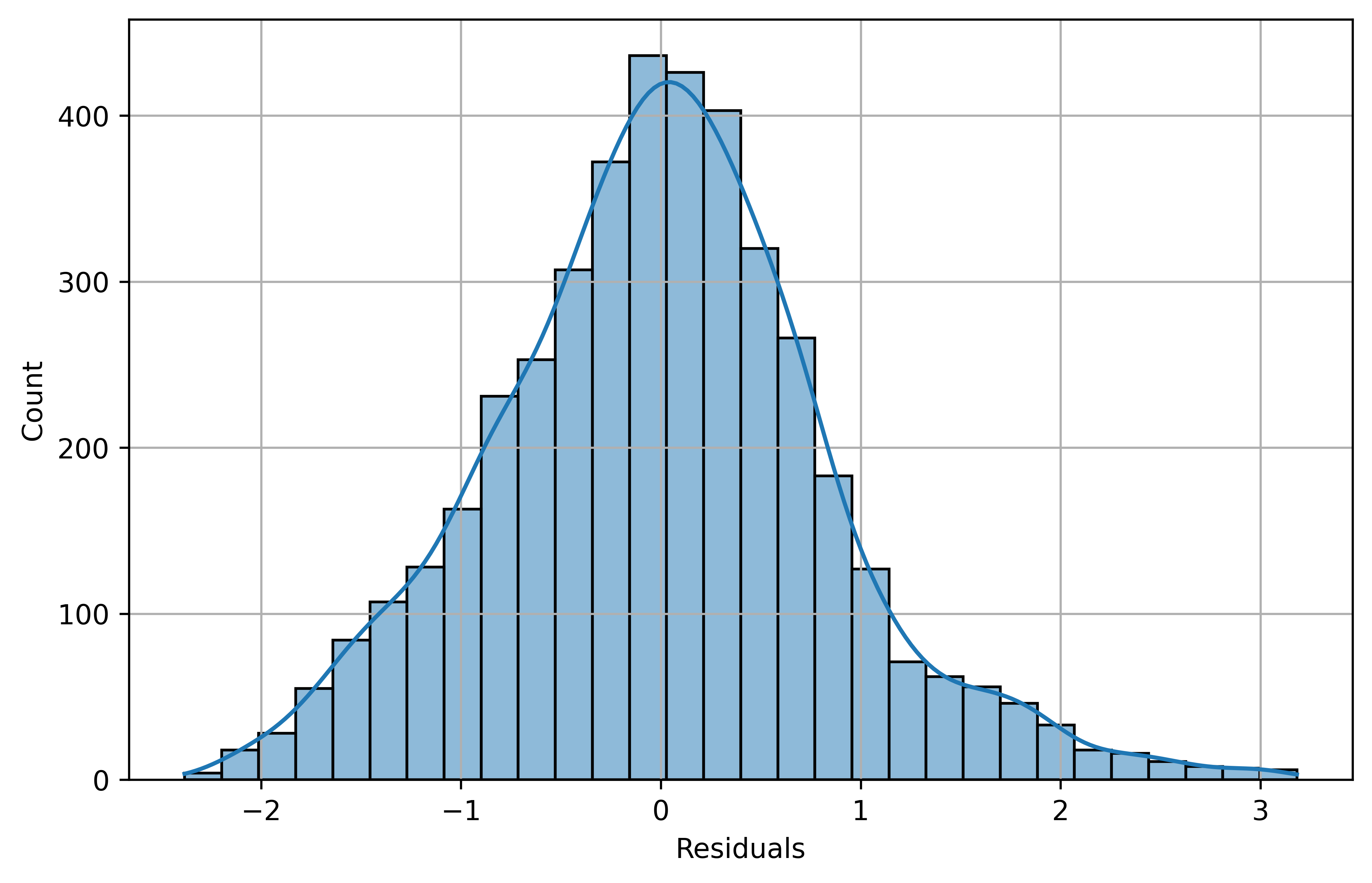

Supplement: S2 File — (ZIP) [file pone.0330211.s002.zip › Fig8_Log_reg_residuals.tif]

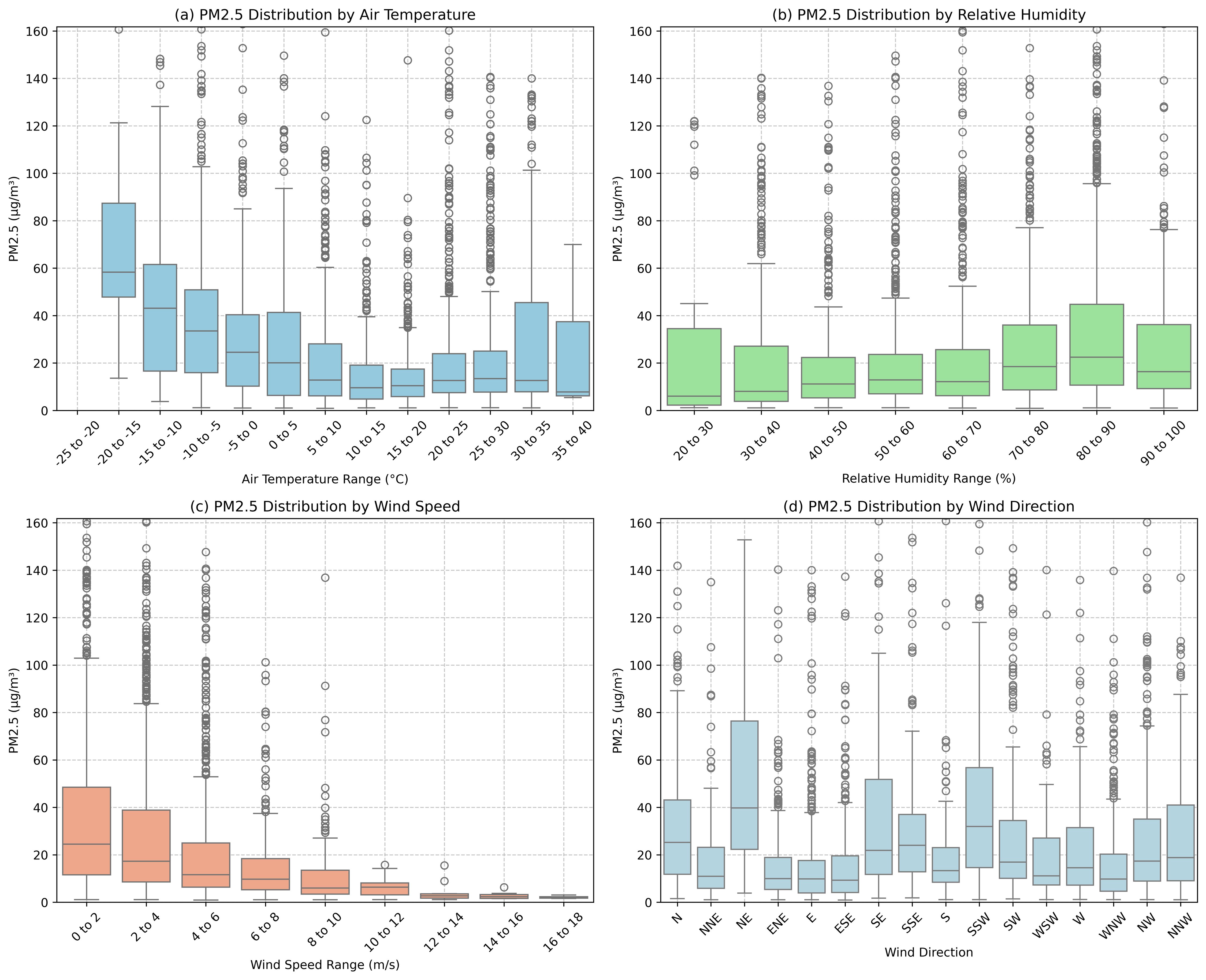

Supplement: S2 File — (ZIP) [file pone.0330211.s002.zip › Fig9_pm25_distribution_by_meteo_extended.tif]

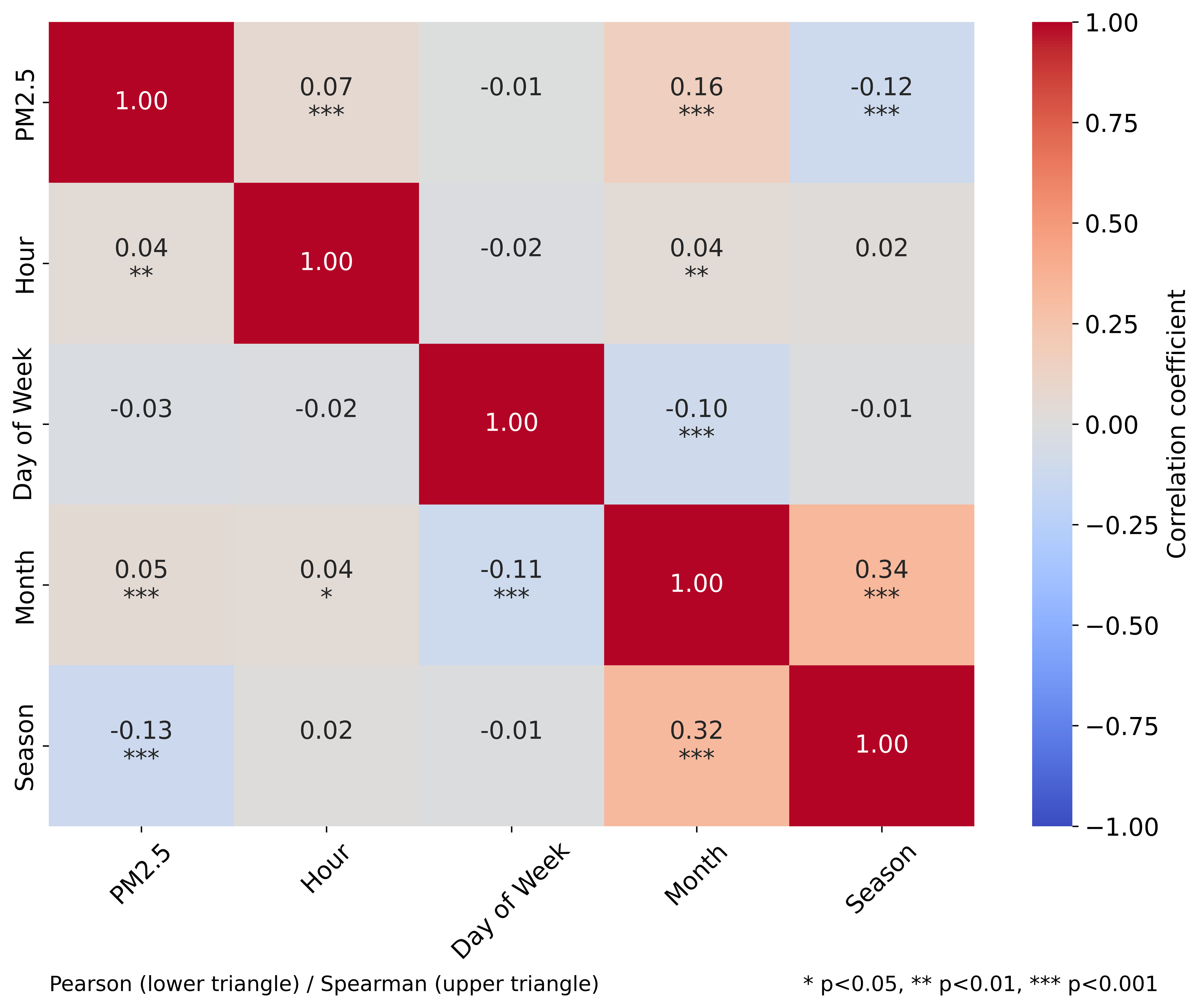

Supplement: S2 File — (ZIP) [file pone.0330211.s002.zip › Fig10_temporal_correlation_heatmap.tif]

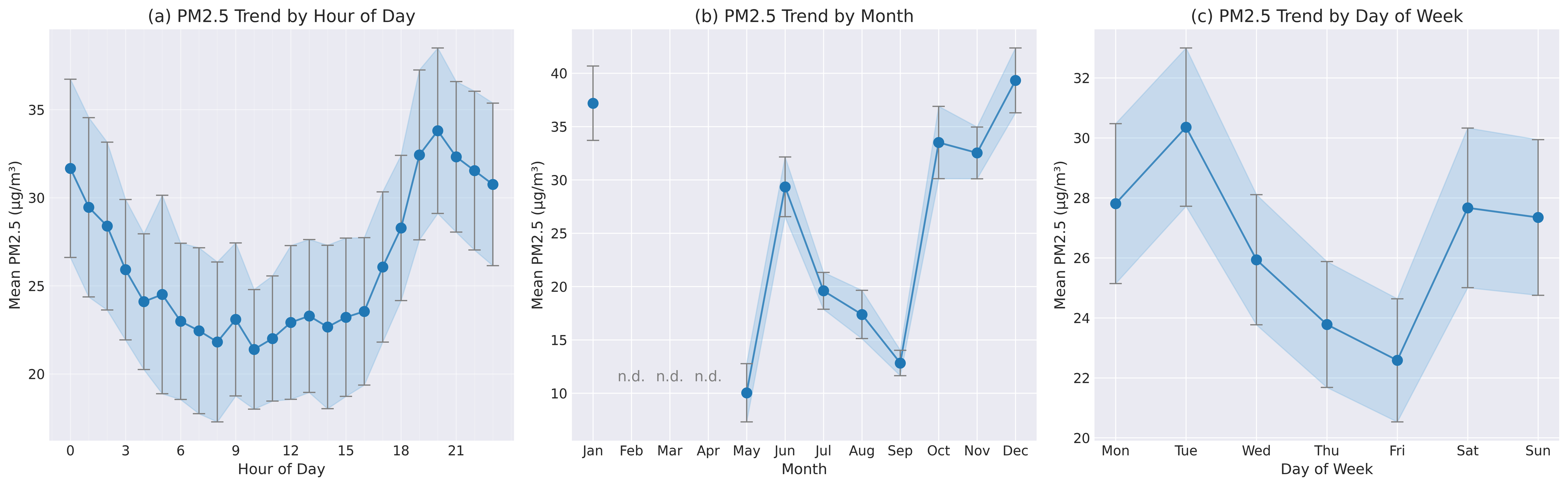

Supplement: S2 File — (ZIP) [file pone.0330211.s002.zip › Fig11_temporal_trends_PM25.tif]

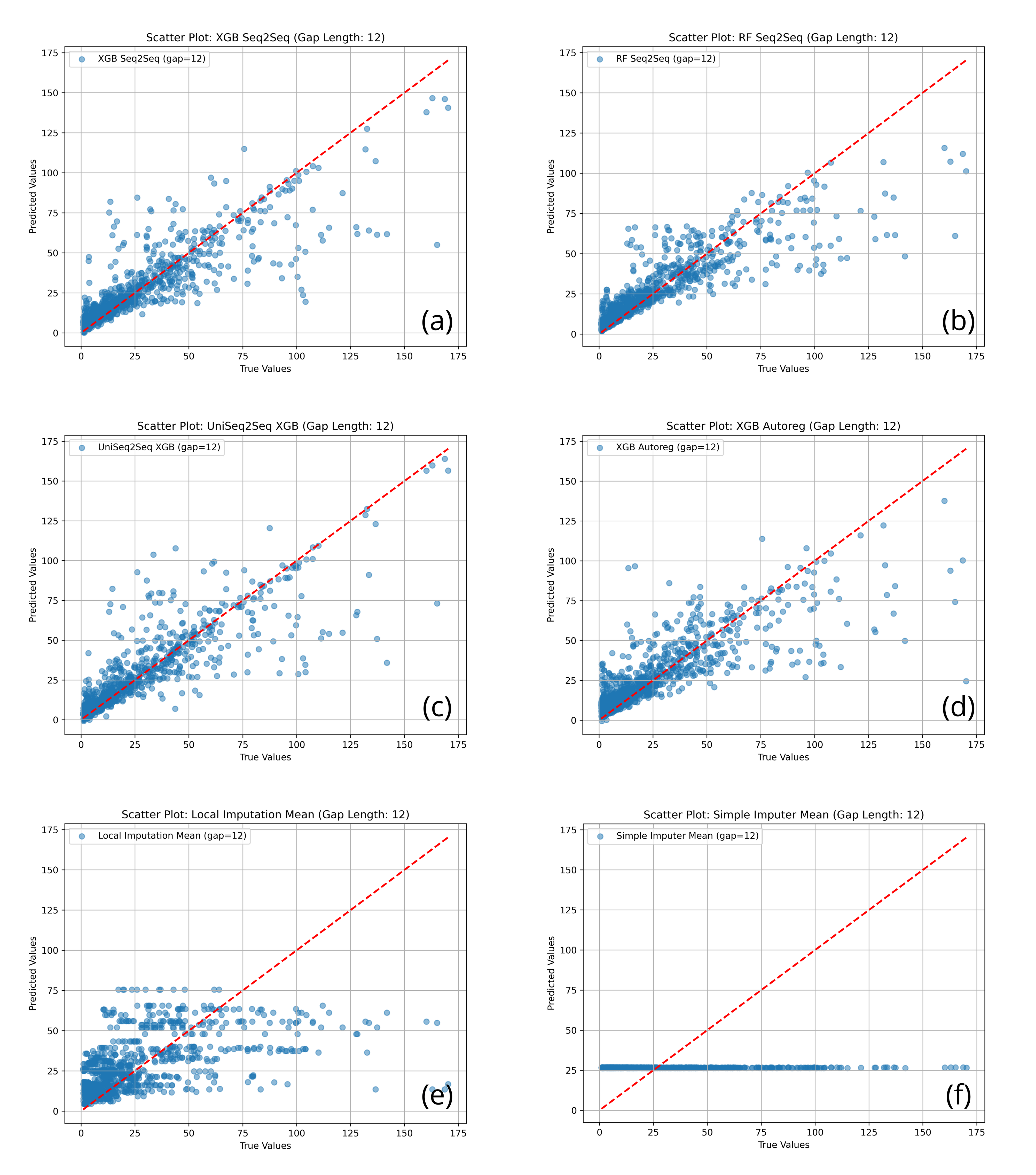

Supplement: S2 File — (ZIP) [file pone.0330211.s002.zip › Fig12_univar_scatter_plots.tif]

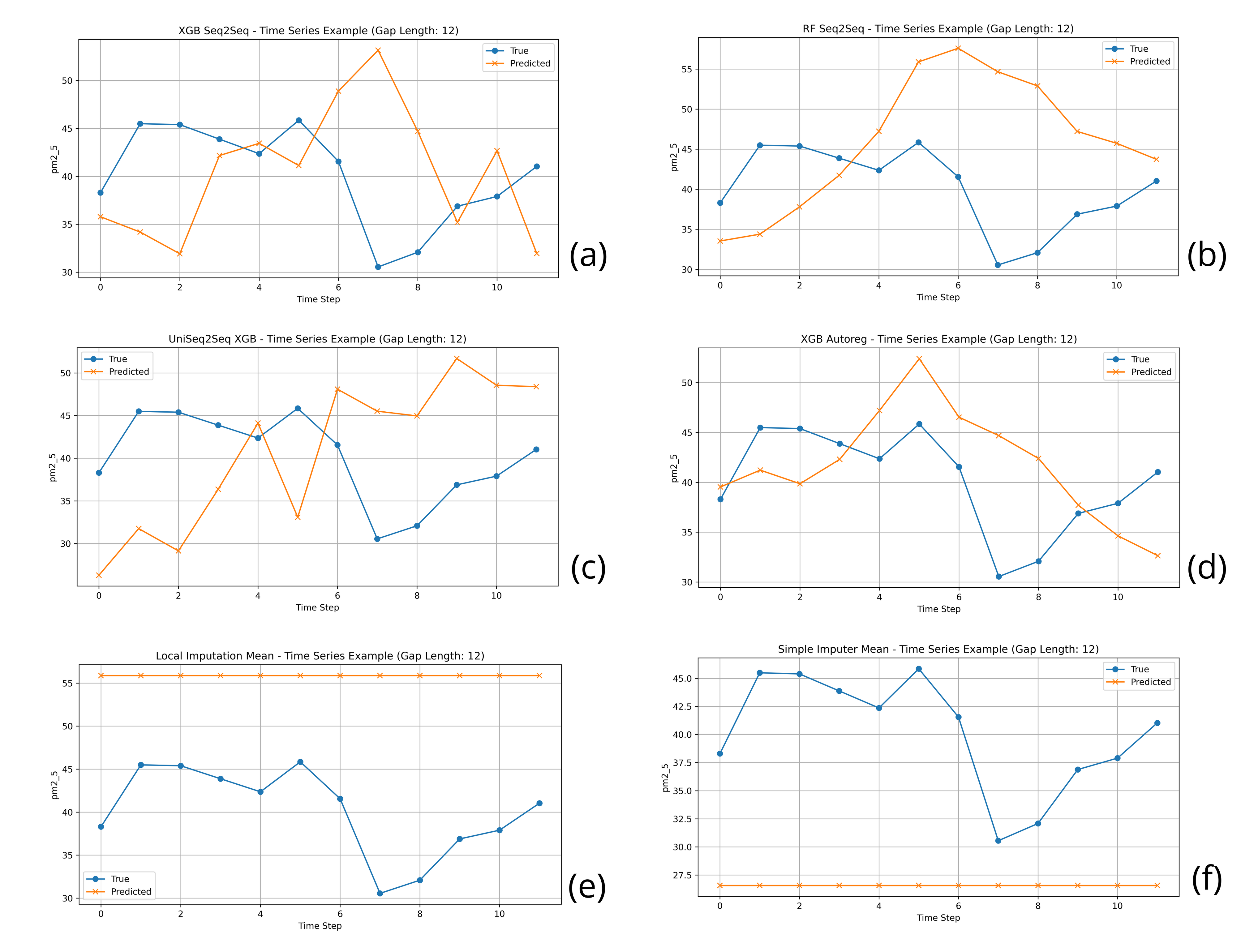

Supplement: S2 File — (ZIP) [file pone.0330211.s002.zip › Fig13_Examples of 12-hour gap filling in PM2.5 time series .tif]

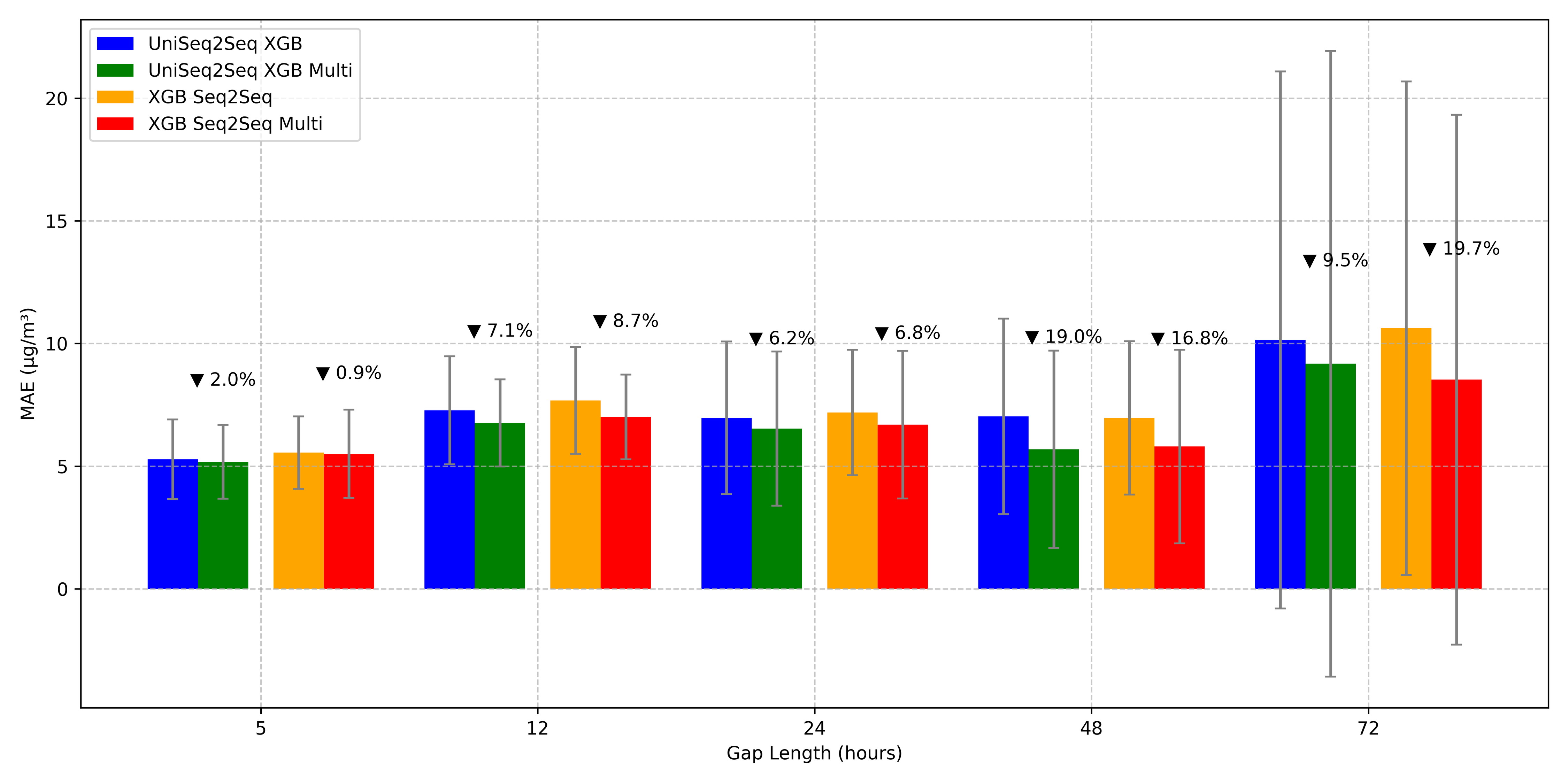

Supplement: S2 File — (ZIP) [file pone.0330211.s002.zip › Fig14_mae_comparison_uni_multi.tif]

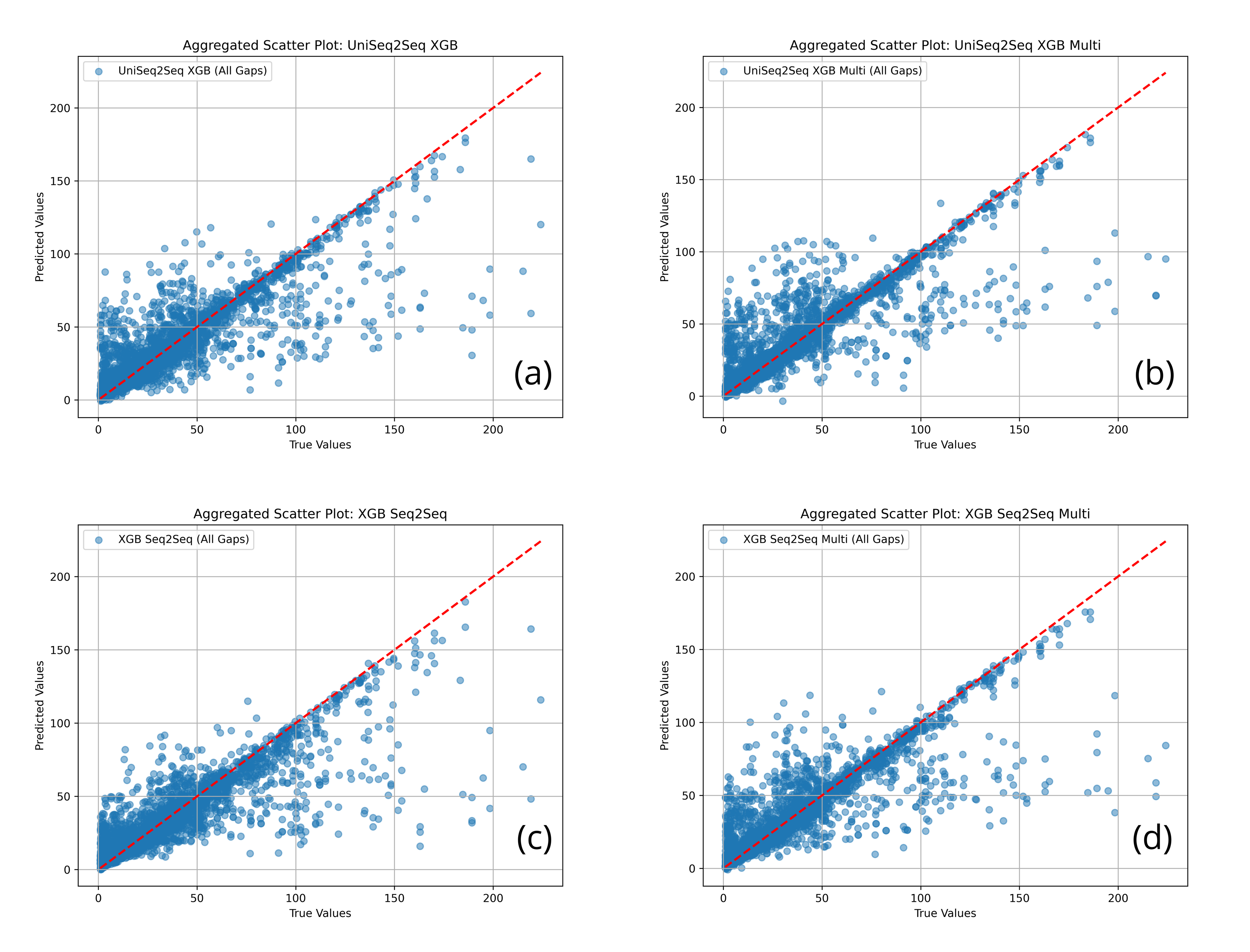

Supplement: S2 File — (ZIP) [file pone.0330211.s002.zip › Fig15_scatter_plots_uni+multi.tif]

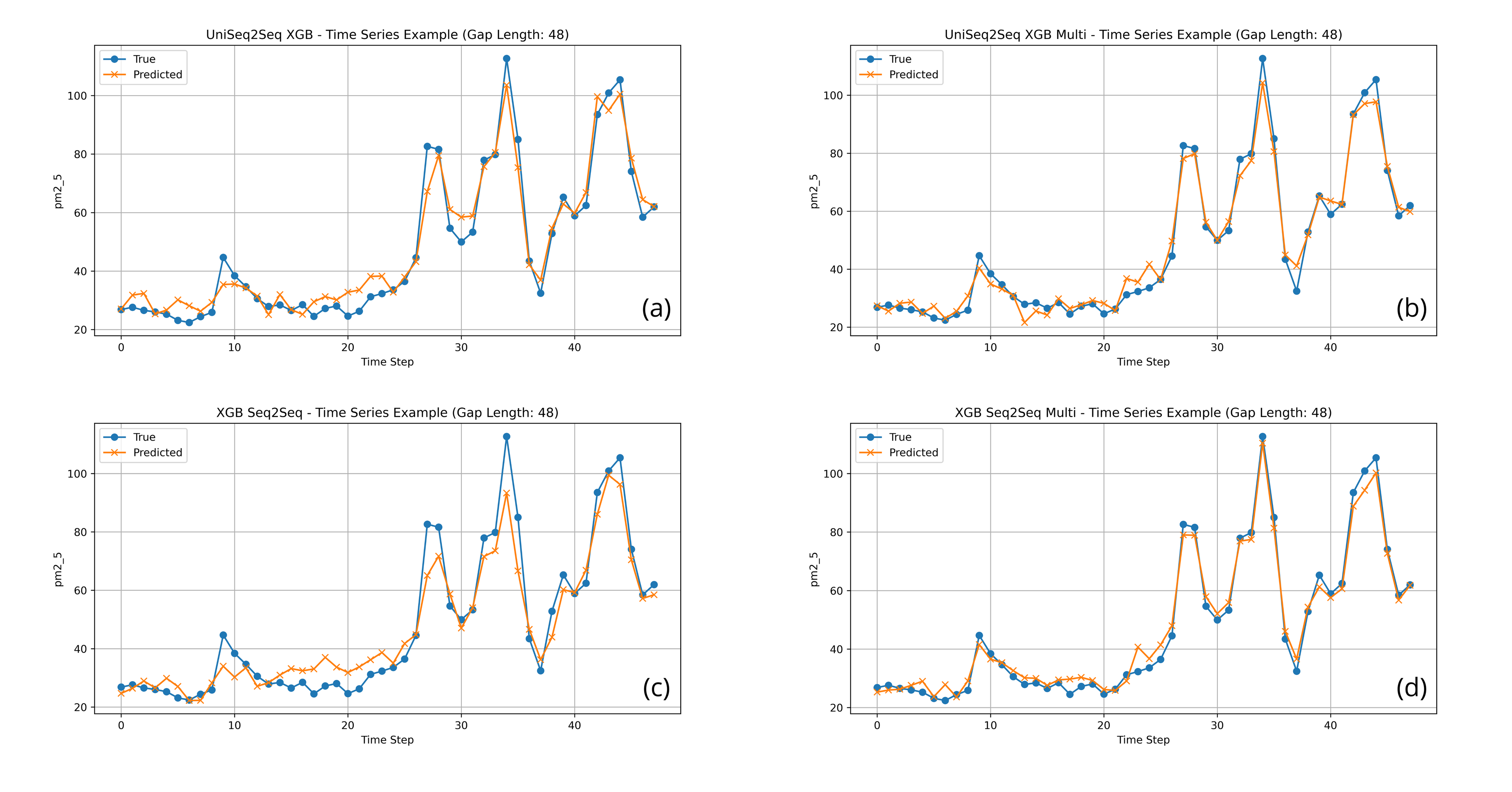

Supplement: S2 File — (ZIP) [file pone.0330211.s002.zip › Fig16_gap_filling_samples uni+multi.tif]

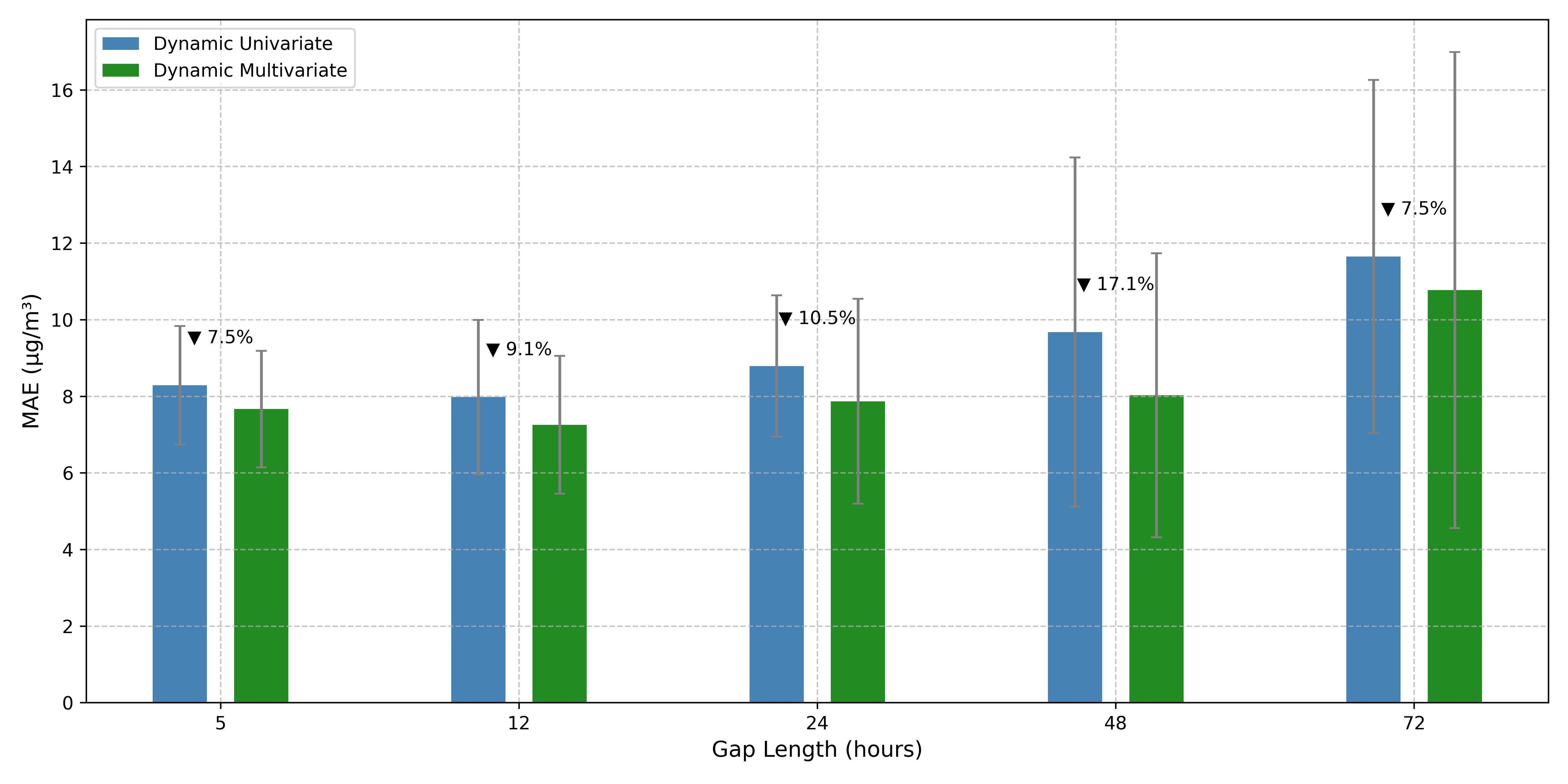

Supplement: S2 File — (ZIP) [file pone.0330211.s002.zip › Fig17_dynamic_models_comparison.tif]

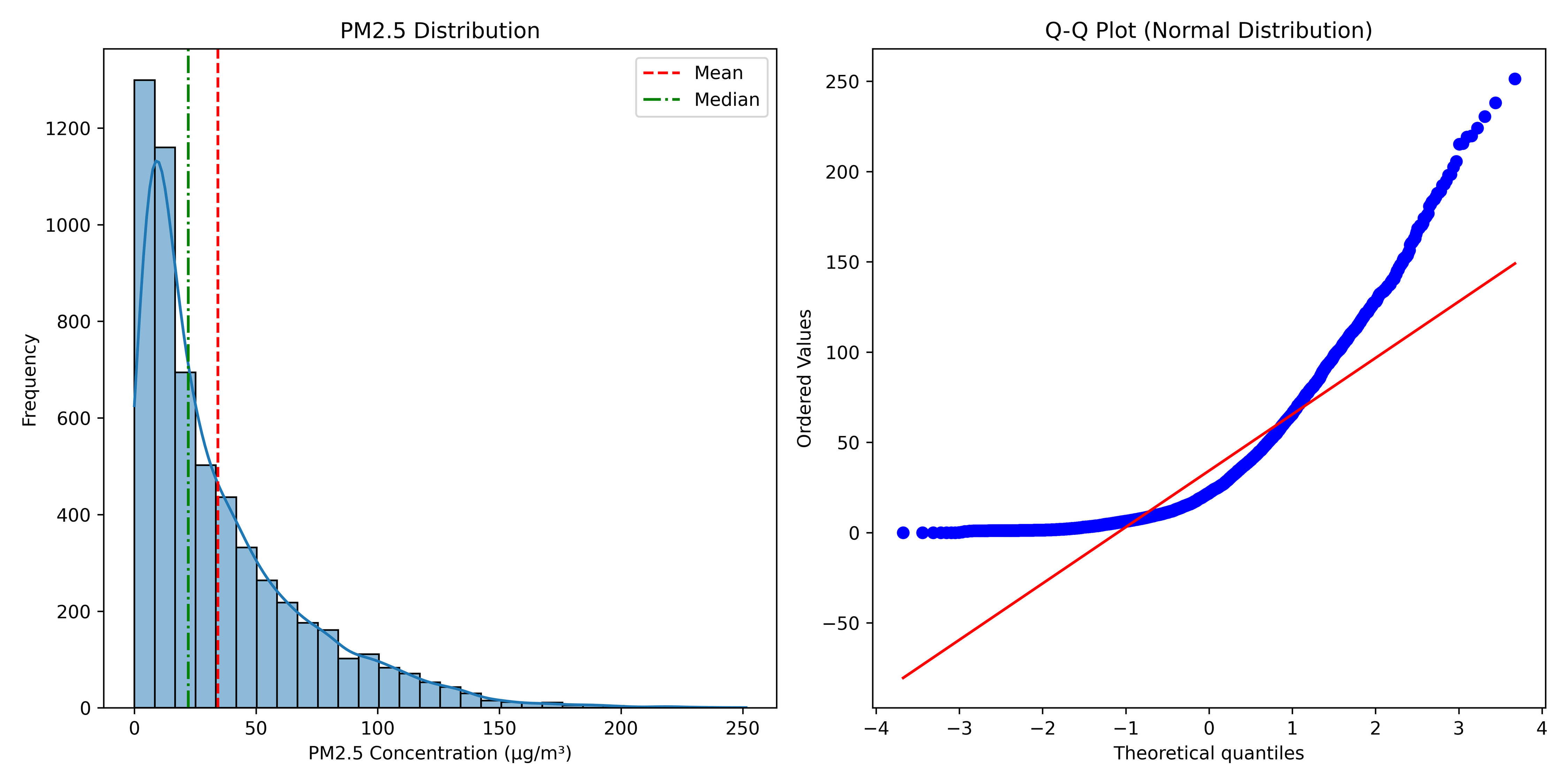

Supplement: S2 File — (ZIP) [file pone.0330211.s002.zip › Fig18_pm25_distribution.tif]

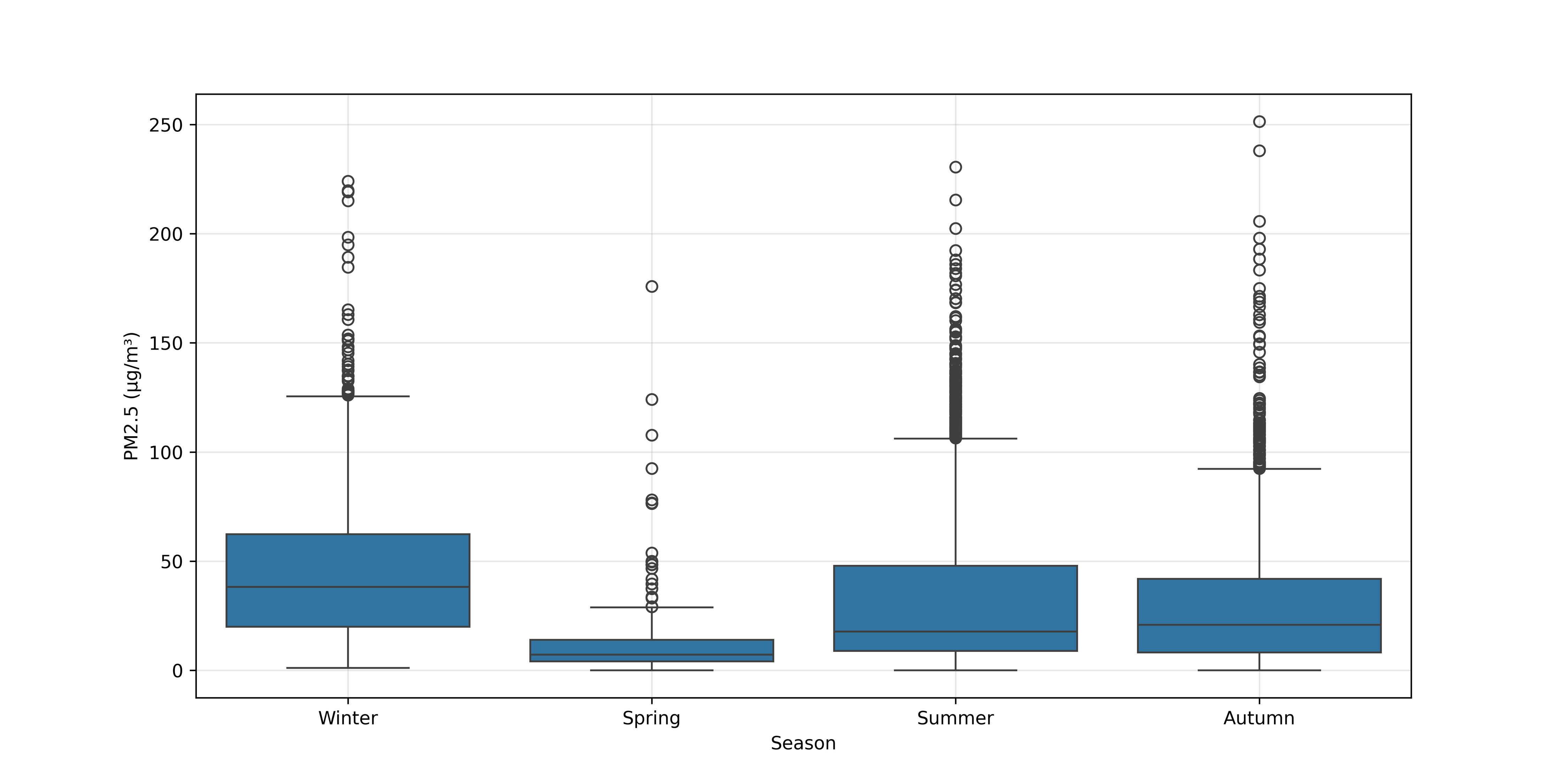

Supplement: S2 File — (ZIP) [file pone.0330211.s002.zip › Fig19_pm25_seasonal_boxplot.tif]

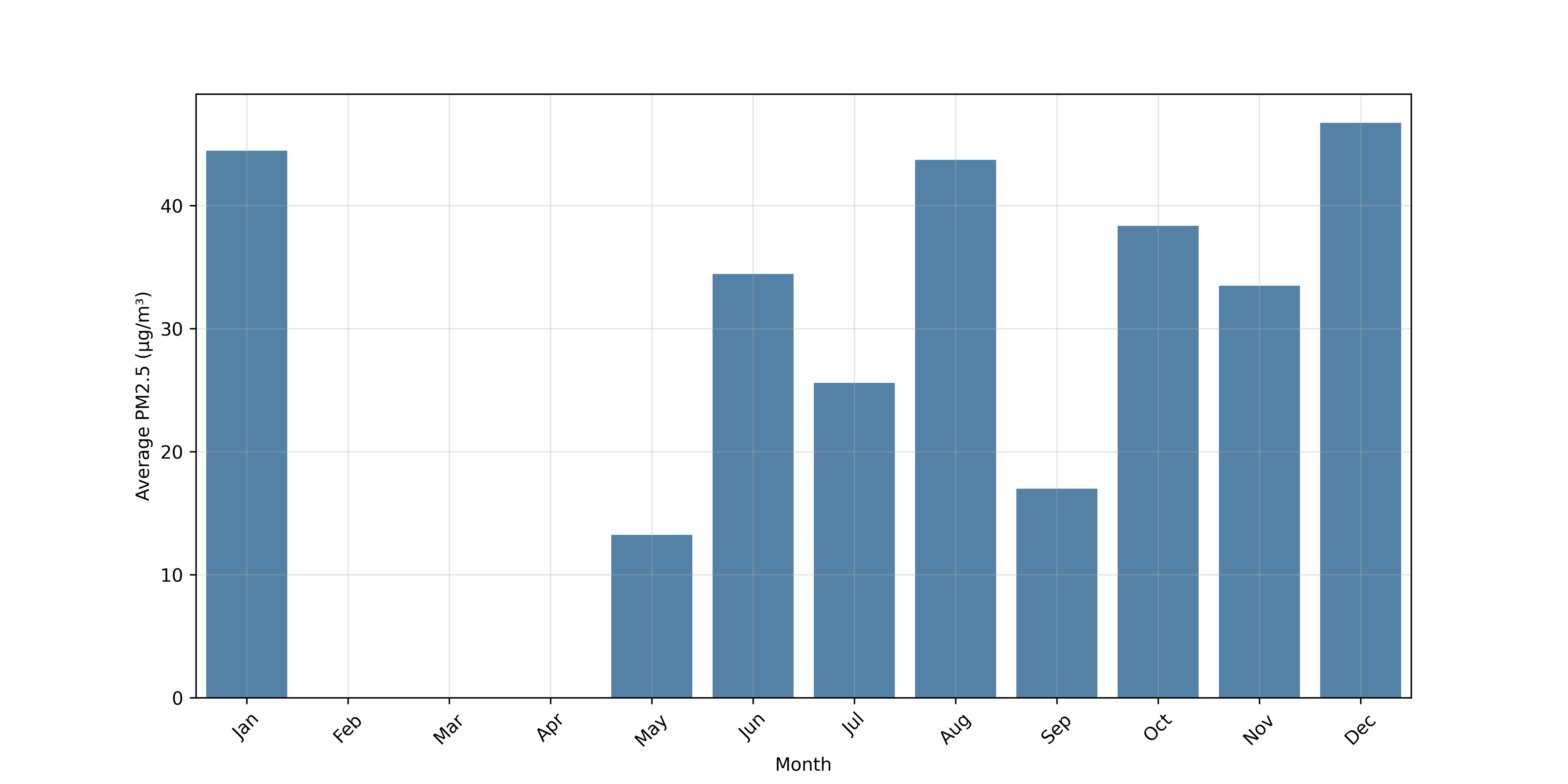

Supplement: S2 File — (ZIP) [file pone.0330211.s002.zip › Fig20_pm25_monthly_averages.tif]

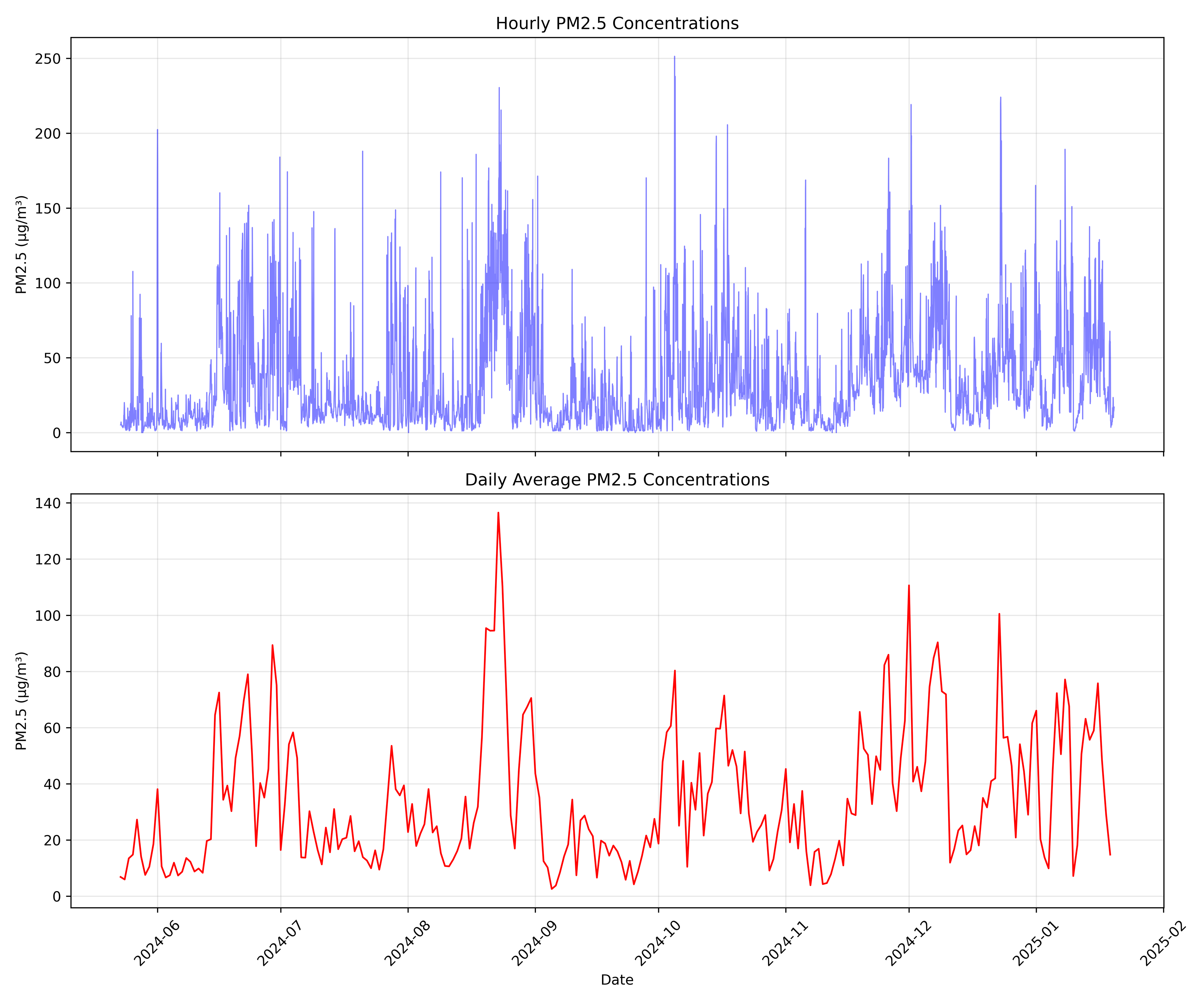

Supplement: S2 File — (ZIP) [file pone.0330211.s002.zip › Fig21_pm25_timeseries.tif]

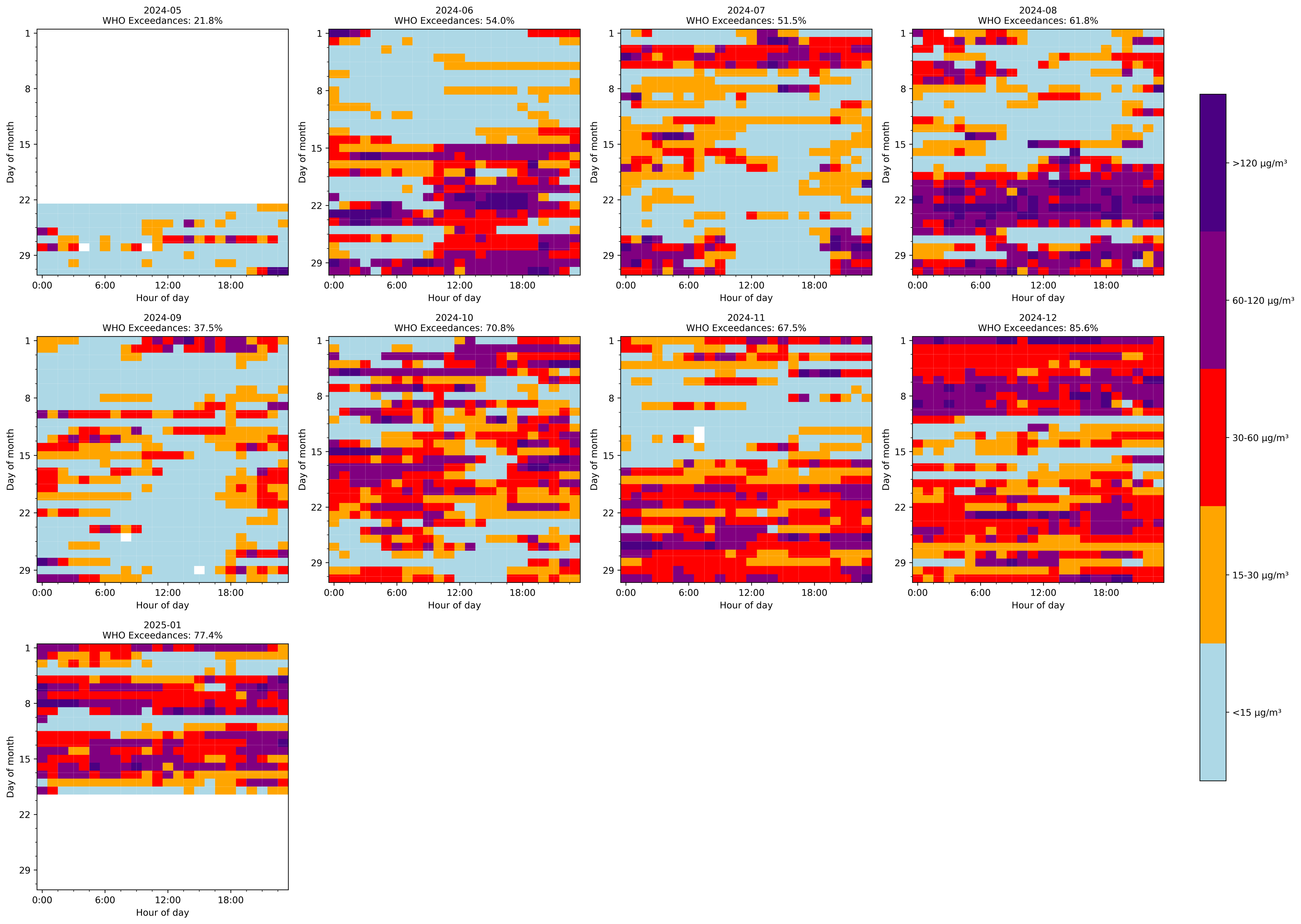

Supplement: S2 File — (ZIP) [file pone.0330211.s002.zip › Fig22_who_exceedances_heatmap.tif]

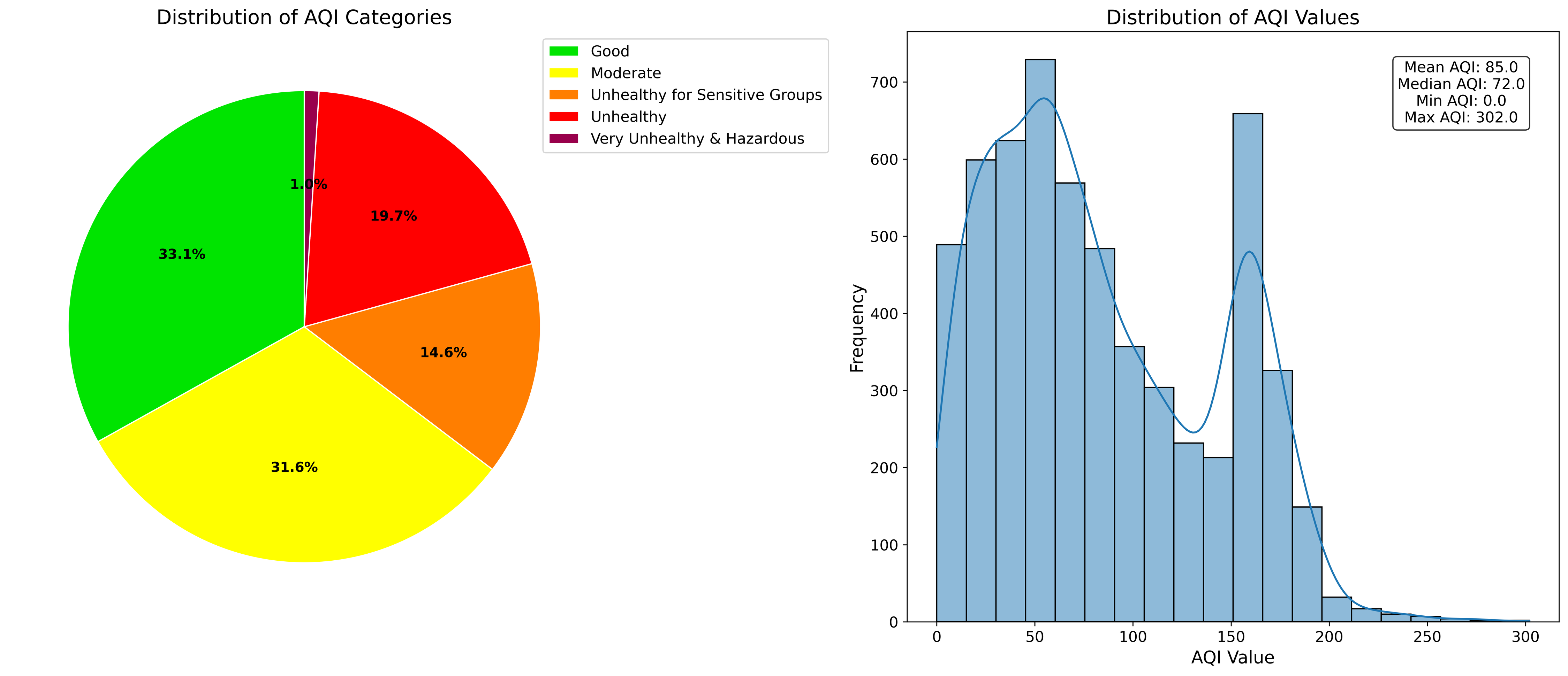

Supplement: S2 File — (ZIP) [file pone.0330211.s002.zip › Fig23_air_quality_index_analysis.tif]

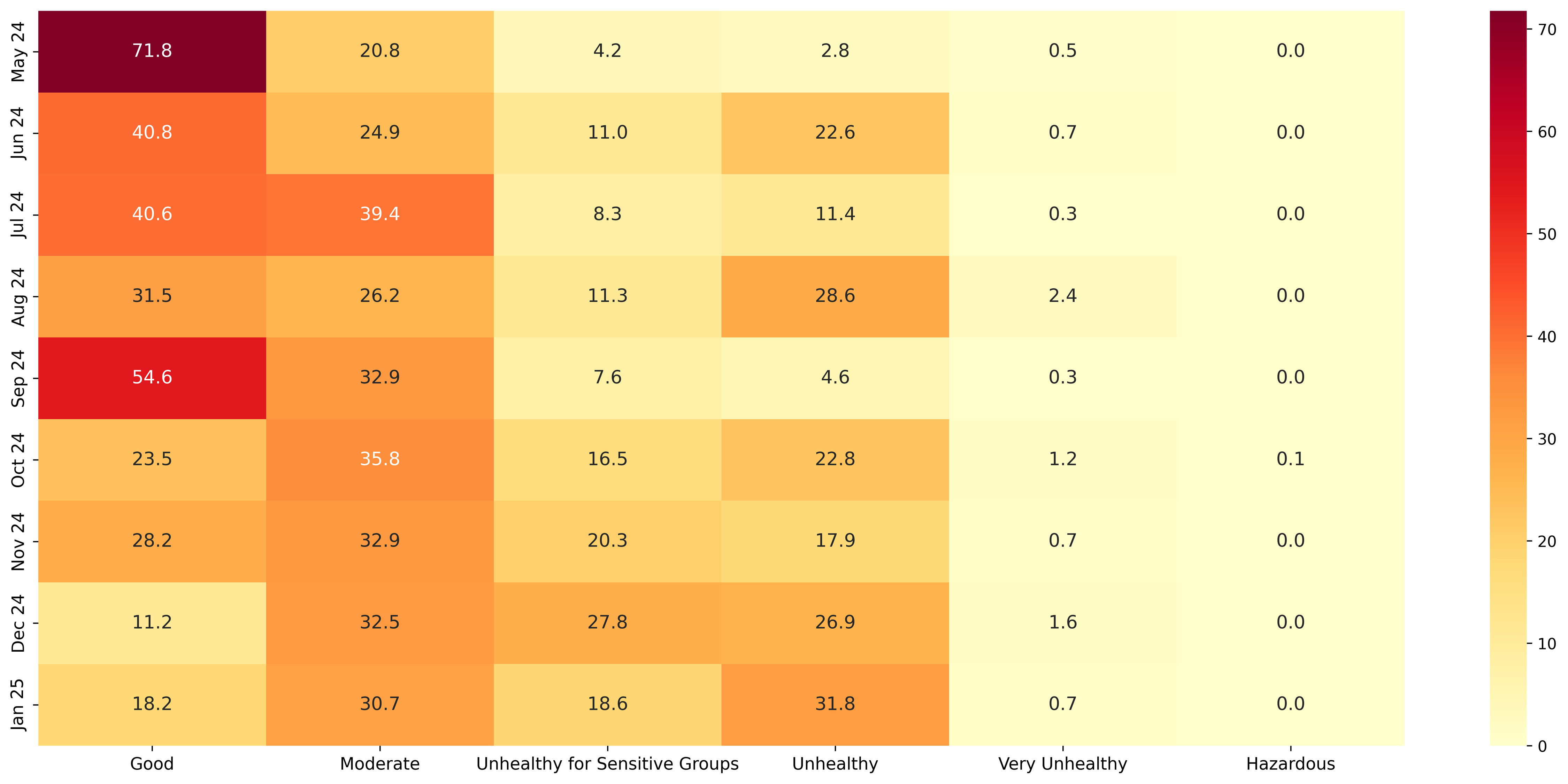

Supplement: S2 File — (ZIP) [file pone.0330211.s002.zip › Fig24_air_quality_index_analysis_monthly_distribution.tif]

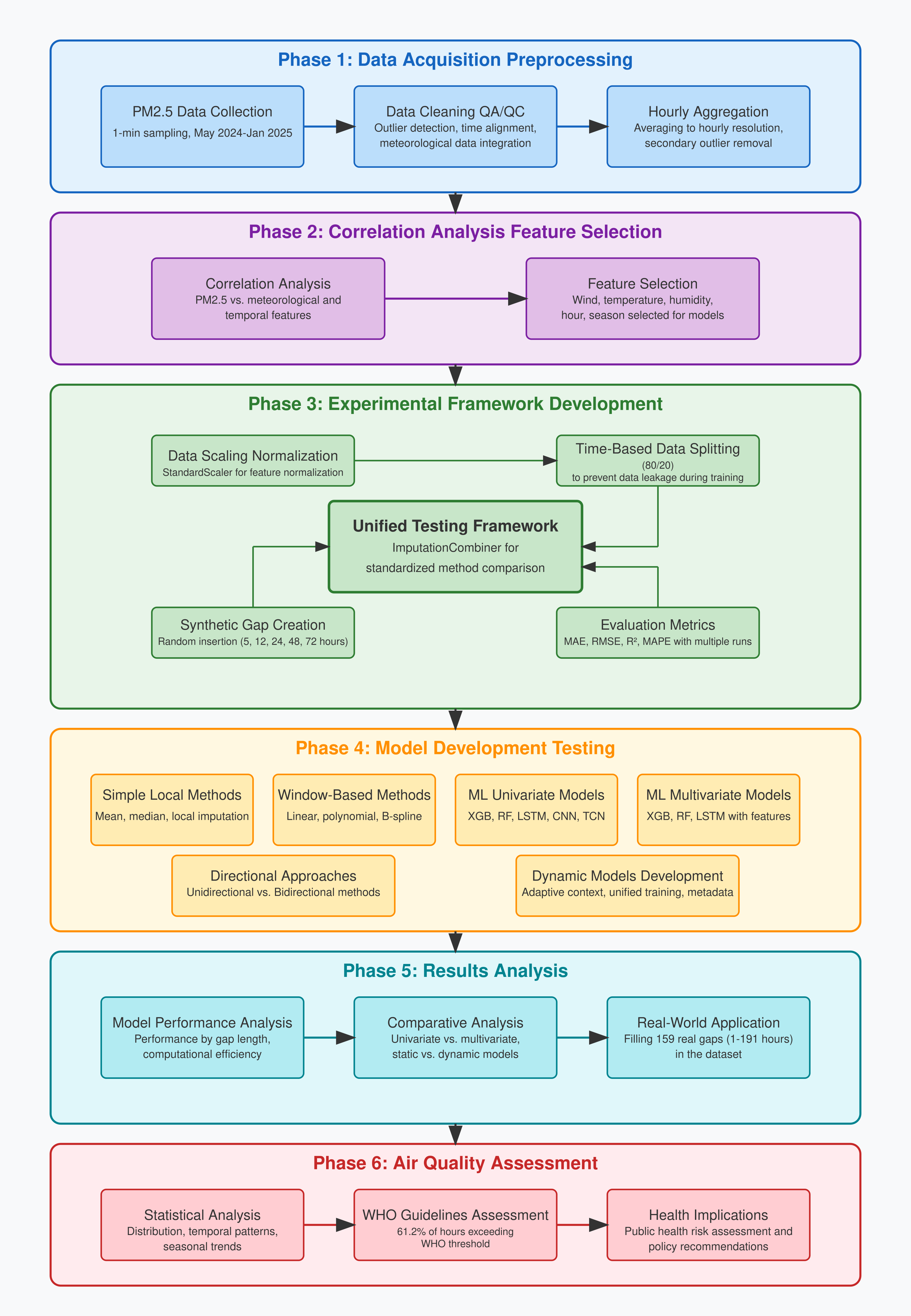

Supplement: S1 Fig — (PNG) [file pone.0330211.s003.png]
